# Forecasting Achievement of Inactive Disease in Juvenile Idiopathic Arthritis with Artificial Intelligence †

**DOI:** 10.3390/children12060741

**Published:** 2025-06-07

**Authors:** Ana I. Rebollo-Giménez, Francesca Ridella, Silvia Maria Orsi, Elena Aldera, Marco Burrone, Valentina Natoli, Silvia Rosina, Alessandro Consolaro, Esperanza Naredo, Angelo Ravelli, Davide Cangelosi

**Affiliations:** 1 UOC Reumatologia e Malattie Autoinfiammatorie, IRCCS Istituto Giannina Gaslini, 16147 Genoa, Italy; 2 Department of Rheumatology, Gregorio Marañón University Hospital, Gregorio Marañón Health Research Institute (IiSGM), 28007 Madrid, Spain; 3 Faculty of Medicine, Autonomous University of Madrid (UAM), 28029 Madrid, Spain; 4 Dipartimento di Neuroscienze, Riabilitazione, Oftalmologia, Genetica e Scienze Materno-Infantili (DINOGMI), Università degli Studi di Genova, 16132 Genoa, Italy; 5 Department of Rheumatology, Joint and Bone Research Unit, Fundación Jiménez Díaz University Hospital, Health Research Institute Fundación Jiménez Díaz (IIS-FJT, UAM), 28040 Madrid, Spain; 6 Direzione Scientifica, IRCCS Istituto Giannina Gaslini, 16147 Genoa, Italy; 7 Unità di Bioinformatica Clinica, Direzione Scientifica, IRCCS Istituto Giannina Gaslini, 16147 Genoa, Italy

**Keywords:** juvenile idiopathic arthritis, pediatric rheumatology, outcome predictors, inactive disease, artificial intelligence, machine learning

## Abstract

**Objective:** to seek for predictors of inactive disease (ID) in juvenile idiopathic arthritis (JIA) with artificial intelligence. **Methods:** The clinical charts of patients seen within 6 months after disease onset between 2007 and 2019 and with follow-up visits at 6, 12, 18, and 24 months were reviewed retrospectively. Sixty-eight potential predictors were recorded at each visit. The primary endpoint was ID at 24 months by 2004 Wallace criteria. Data obtained from diverse combinations of visits were examined to identify the best forecasting model. After pre-processing, the cohort was divided into training (50%) and testing (50%) cohorts. Multivariate time series forecasting, coupled with the Random Forest method, was used to train the machine learning (ML) forecasting model. Predictive performance was assessed through the Matthews correlation coefficient (MCC). **Results:** A total of 414 patients were included. The best performance in predicting ID at 24 months in the training cohort was provided by the 0–12 months interval (MCC = 0.68). In the testing cohort, the same ML model confirmed a high forecasting performance (MCC = 0.65). Assessment of feature importance and impact analysis showed that the most relevant predictor of ID was the physician’s global assessment (PhGA), followed by the count of active joints (AJC). **Conclusions:** PhGA and AJC values over the first 12 months were the strongest predictors of ID at 24 months. This finding highlights the importance of regular quantitative assessment of disease activity by the caring physician in monitoring the course of the patient toward achievement of complete disease quiescence.

## 1. Introduction

Juvenile idiopathic arthritis (JIA) is a chronic rheumatic disease characterized by prolonged synovial inflammation that may lead to progressive joint damage and disability. Permanent changes may also develop in extraarticular organs/systems, such as the eye (as a complication of chronic anterior uveitis), or may result from side effects of medications, especially systemic glucocorticoids [1]. This morbidity may have a marked impact on the quality of life of patients and their families [2,3].

Over the past two decades, there has been a major advance in the management of JIA, which has made remission an achievable goal for the vast majority of patients [4]. Complete disease quiescence is regarded as the ideal therapeutic objective because its attainment may prevent long-term articular and extra-articular damage and functional impairment [5]. The recommendations for the treat-to-target strategy in JIA have set inactive disease (ID) as the primary target for treatment [6].

Predicting the achievement of ID in JIA is vital to guiding and optimizing treatment decisions. It would be desirable to differentiate early in the course of the illness those patients who are likely to have potentially destructive arthritis from those with self-limiting or non-erosive disease. A better understanding of disease trajectories may facilitate early intervention strategies tailored to individual patient characteristics. However, JIA is a heterogeneous condition which varies widely from one patient to another in terms of disease manifestation, course, phenotype, and response to therapies. Although several prognostic factors have been identified across the various categories of JIA [7], reliable and consistent predictors of therapeutic response and outcome are not yet available for use in routine clinical practice. Notably, most studies have focused on the search for baseline predictors, whereas the development of longitudinal prediction models has been seldom attempted.

In recent years, artificial intelligence (AI) has emerged as a powerful tool that can facilitate screening, diagnosis, monitoring, risk assessment, prognosis determination, achievement of optimal treatment outcome, and de novo drug discovery for patients with rheumatic disorders [8,9,10,11]. Investigation of the potential applications of AI, including machine learning (ML) and deep learning techniques, is an exponentially growing field in medicine and healthcare. It has been suggested that incorporation of these methods can be critical to providing high-quality care to patients with chronic rheumatic diseases who lack an optimal treatment [12]. Notably, AI technologies are potentially well suited for constructing longitudinal models of ID prediction in JIA due to their ability to analyze complex, multivariate, and temporal data. They can help identify patterns and trends across multiple time points, which is essential for tracking disease progression. Their capacity to capture non-linear relationships and to select the most relevant features enhances both the accuracy and interpretability of predictions. These properties make AI a valuable tool for personalized and dynamic modeling of disease outcomes in JIA. Against this background, the primary aim of this study was to develop and validate a multivariable forecasting model of ID in JIA through the use of longitudinal data.

## 2. Materials and Methods

### 2.1. Study Design and Patient Selection

The clinical charts of all consecutive patients with JIA as defined by the International League of Associations for Rheumatology (ILAR) criteria [13], who were first seen at the Gaslini Institute of Genoa, Italy, in the first 6 months following disease onset between 2007 and 2019 and who had a follow-up visit with available information at 6, 12, 18, and 24 months after initial evaluation were reviewed retrospectively.

### 2.2. Clinical Assessment

Baseline information included sex, age, disease duration from occurrence of the first symptoms consistent with JIA, and an ILAR category. For simplicity, we grouped patients with different ILAR categories into four “functional” disease phenotypes: systemic arthritis, polyarthritis (including extended oligoarthritis and rheumatoid factor, RF-positive and RF-negative polyarthritis), oligoarthritis (including persistent oligoarthritis), and other arthritis (including enthesitis-related arthritis, psoriatic arthritis and undifferentiated arthritis). The following data were extracted for each study visit: physician global assessment (PhGA) of overall disease activity using a 21-numbered circle numerical rating scale (NRS), ranging from 0 (=no activity) to 10 (=maximum activity) [14]; active joint count (AJC), assessed in 73 joints [15]; type of affected joints; presence of active systemic manifestations (fever, rash, hepatosplenomegaly, lymphadenopathy, serositis); and presence of active uveitis by the examining ophthalmologist. A joint was defined as active if it displayed swelling or, in case swelling was absent or not detectable (as in the case of cervical spine or hip), pain/tenderness plus restricted motion [16]. Laboratory tests included the erythrocyte sedimentation rate (ESR) and C-reactive protein (CRP). Patients were considered ANA-positive if they had a minimum of two positive ANA test results, obtained at least three months apart during follow-up, using indirect immunofluorescence on Hep-2 cells at a titer of ≥1:160 [17]. The medications administered between study visits were recorded.

Data collection was carried out by five pediatric rheumatology fellows (AIRG, SO, FR, EA, and VN), with oversight provided by an experienced investigator (AR).

### 2.3. Study Endpoint

The study endpoint was the achievement of ID at 24 months from the baseline evaluation. The state of ID was defined according to the 2004 Wallace criteria, i.e., as joint(s) with active arthritis, no systemic manifestations attributable to JIA, no active uveitis, normal acute-phase reactants, and PhGA indicating no disease activity (defined as a score of 0 on the 0–10 VAS)  [18]. However, in a subset of patients, the full application of the Wallace criteria was not possible due to missing PhGA data. In visits where this parameter was unavailable, but all other Wallace criteria were fulfilled, inactive disease status was inferred, following the approach adopted in previous studies [19,20], by reviewing the patient chart until a consensus by two investigators was reached (AIRG and VN). To support this assessment, the attending physician who had initially evaluated the patient during the visit was independently asked to review their clinical notes and verify the state of inactive disease. Any discrepancies between the treating physician and the investigators were resolved through consensus involving the two investigators and a senior author (AR) [21].

### 2.4. Statistical Analysis

Descriptive statistics were first employed to provide an overview of the baseline characteristics of the study population. Continuous variables were summarized using medians and interquartile ranges (1st–3rd quartiles), while categorical variables were described by their absolute frequencies and corresponding percentages.

### 2.5. Machine Learning Analysis

The Last Observation Carried Forward (LOCF) method and subsequently the Baseline Observation Carried Forward (BOCF) method, implemented in the Pandas python package [22], were employed for imputing missing values. Patients with remaining missing values after imputation were excluded from the analysis. The mlforecast package, version 0.15.0, was utilized to train the multivariate time series model and generate forecasts. Exogenous features with available values at 24 months were used for forecasting. The recursive method was employed with the horizon parameter set to 1.

Hyperparameter tuning was conducted using the Optuna package [23] with a TPESampler seed set to 10. A total of 100 Optuna trials were executed to optimize the model’s parameters. The parameter search space for Random Forests included n_estimators ranging from 2 to 500, max_depth from 2 to 32, min_samples_split from 2 to 10, min_samples_leaf from 3 to 10, and the random_state set to 0. Random Forests [24], penalized logistic regression, K-nearest neighbors (KNN), and a support vector machine (SVM) [25] were used in combination with  MLforecast for binary classification. To ensure an unbiased assessment of model performance, the dataset was randomly split into training (50%) and testing sets (50%). The training set was used to build forecast models, while the testing set was used to estimate prediction performance. The Matthews correlation coefficient (MCC) was chosen as the metric to assess model performance. The MCC ranged from −1 to +1, where +1 indicates perfect prediction and −1 indicates imperfect prediction. The scale for evaluating prediction performance was defined as follows: 0 ≤ MCC ≤ 0.19 (very low), 0.2 ≤ MCC ≤ 0.39 (low), 0.4 ≤ MCC ≤ 0.59 (moderate), 0.6 ≤ MCC ≤ 0.79 (high), and 0.8 ≤ MCC ≤ 1.0 (very high) [26]. Feature importance in the training set for Random Forests was estimated using the feature_importances_attribute of the fitted RandomForestClassifier function, calculated as the mean and standard deviation of the impurity decrease across each tree in the trained model. To expedite model training, parallelization was implemented using the Dask package [27]. Feature impact in the testing set was estimated using shap package by setting up shap via the Explainer function [28].  Beeswarm plots were utilized to visualize the results for global explainability of the model. Penalized logistic regression, KNN, and SVM model were implemented in scikit-learn package version 0.15 [25].

### 2.6. Patients and Public Involvement

This study was conducted in accordance with the ethical principles outlined in the Declaration of Helsinki. All parents or guardians, or patients themselves if age appropriate, were routinely asked to provide written consent to the use of patients’ clinical data for research purposes during the first observation at the study center. The study protocol was approved by the Ethics Committee of Regione Liguria (Genoa, Italy) under protocol number 642/2022—DB id 12828, dated 16 June 2023 [21].

## 3. Results

### 3.1. Patient Population and Dataset Creation

**Figure 1:**
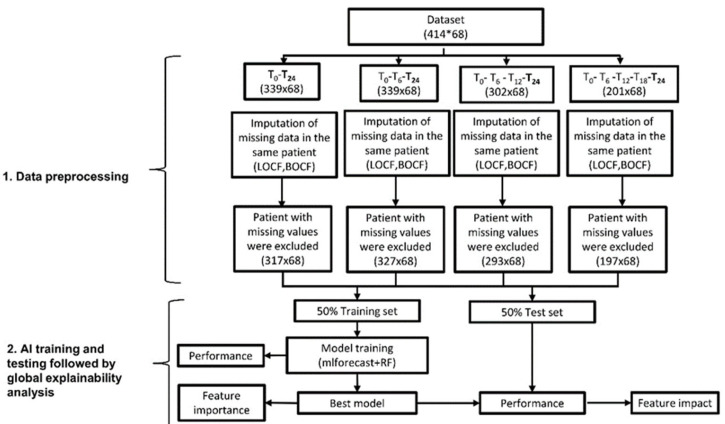
Workflow of the analysis illustrating data pre-processing, AI training, and testing, followed by a global explainability analysis. LOCF = Last Observation Carried Forward. BOCF = Baseline Observation Carried Forward; RF = Random Forests; AI = artificial intelligence.

A total of 414 patients, whose main clinical features at study entry are shown in Table 1, were included in this study. Table 2 reports the cumulative frequency of the medications administered during the 24 months of follow-up. Our patient population, which is largely represented by children with oligoarticular onset disease, and by our policy of administering intra-articular glucocorticoid therapy in all affected joints as first-line treatment in most children with either oligoarthritis or with polyarthritis and the predominant involvement of large joints. The majority of children who were not receiving any DMARDs were those who experienced sustained remission after such a therapeutic approach. Figure 1 illustrates the schematic representation of the analysis workflow. Patients without a follow-up visit at 24 months, which precluded assessment of the study endpoint, were excluded from the analysis. To evaluate the ability of clinical features to forecast the ID status at 24 months and to identify the earliest follow-up visit capable of accurately predicting the ID status, the dataset was divided into four distinct subsets: T0-T24, T0-T6-T24, T0-T6-T12-T24, and T0-T6-T12-T18-T24. These subsets comprised 339, 339, 302, and 201 patients, respectively, who were retained for subsequent analyses. A total of 68 clinical features, listed in the Supplementary Table S1, were assessed at each time point for their predictive ability. Supplementary Table S2 reports the number of patients for each feature, time point, and feature value. After data imputation and subsequent exclusion of patients with remaining missing values, 317, 327, 294 and 197 patients, respectively, were retained in each of the above datasets for further analysis.

### 3.2. Forecast of the State of ID at 24 Months

Each dataset was randomly split into training and testing sets to train the time series forecasting model and generate independent forecasts for the testing set. The MLforecast method, coupled with the RandomForests algorithm, exhibited a heterogeneous forecasting performance in both the training and the testing sets across different datasets, as summarized in Table 3.

The following most accurate Random Forests models were identified for each dataset:

T0-T6-T12-T18-T24: ‘n_estimators’: 160, ‘max_depth’: 32, ‘min_samples_split’: 4, ‘min_samples_leaf’: 10.
T0-T6-T12-T24: ‘n_estimators’: 437, ‘max_depth’: 30, ‘min_samples_split’: 10 ‘min_samples_leaf’: 4.
T0-T6-T24: ‘n_estimators’: 262, ‘max_depth’: 20, ‘min_samples_split’: 5, ‘min_samples_leaf’: 5.
T0-T24: ‘n_estimators’: 157, ‘max_depth’: 11, ‘min_samples_split’: 6, ‘min_samples_leaf’: 6.

In the training set, the performance was high for both the T0-T6-T12-T18-T24 and T0-T6-T12-T24 datasets, which achieved MCC scores of 0.70 and 0.68, respectively. The T0-T6-T24 dataset showed a moderate MCC of 0.57. However, the forecasting performance was 0.0 for the T0-T24 dataset, indicating that the model trained solely on baseline features was unable to accurately predict the ID status at T24. Based on the training set performance, the forecasting model developed for the T0-T6-T12-T24 dataset demonstrated the best balance between performance and early prediction capability. In the testing set, the T0-T6-T12-T24 dataset achieved the highest MCC of 0.65, indicating its superior effectiveness in predicting the ID status at 24 months. Lower or comparable MCC were obtained with penalized logistic regression, KNN and SVM models on the training and test sets (Supplementary Table S3).

**Figure 2:**
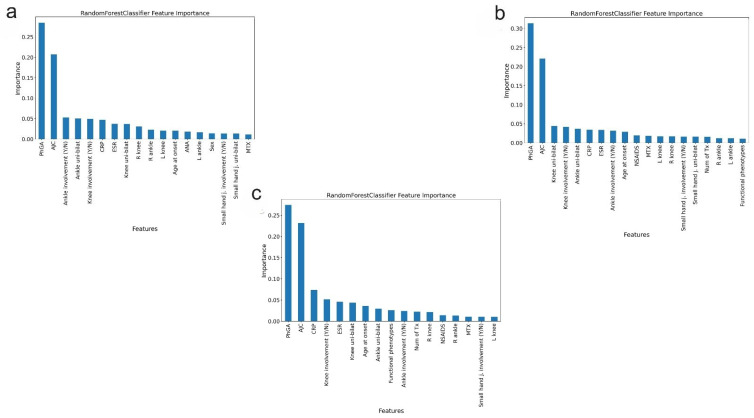
Feature importance in the training set (global explainability): Random Forests. (**a**) Random Forests using the T0-T6-T24 dataset. (**b**) Random Forests using the T0-T6-T12-T24 dataset. (**c**) Random Forest using the T0-T6-T12-T18-T24 dataset. The inclusion of features was restricted to those with an importance greater than 0.01. The most relevant predictors of ID status at 24 months were PhGA and AJC. ID = inactive disease; PhGA = physician global assessment; AJC = active joint count; CRP = C-reactive protein; (Y/N) = Yes/No. Uni-bilat = unilateral–bilateral; ESR = Erythrocyte Sedimentation Rate; R = Right; NSAIDS = non-steroidal anti-inflammatory drugs; L = left; MTX = methotrexate; IAC = intraarticular corticosteroids, j = joint, GS = glucocorticoids, ETA = etanercept; ANA = antinuclear antibody; RF = rheumatoid factor; TMJ = temporomandibular joint.

**Figure 3:**
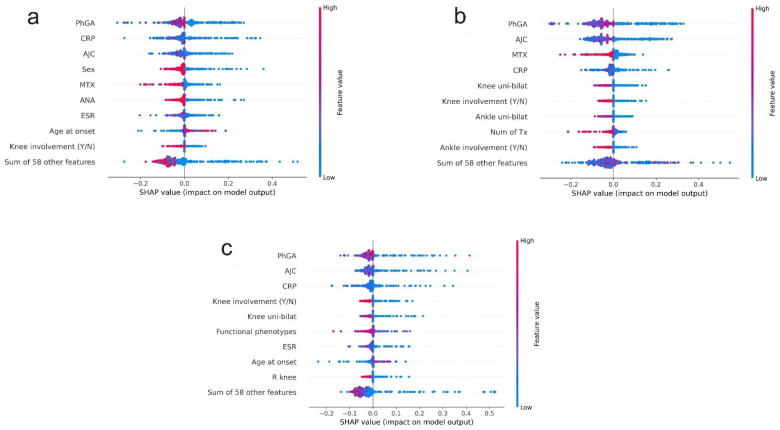
Feature impact on the test set (global explainability): SHAP values. (**a**) shows the SHAP values using the T0-T6-T24 dataset. (**b**) the SHAP values using the T0-T6-T12-T24 dataset. (**c**) the SHAP values using the T0-T6-T12-T18-T24 dataset. AJC and CRP were the features with the highest predictive impact for the T0-T6-T24, T0-T6-T12-T24 and T0-T- T12-T18-T24 datasets. SHAP values = SHapley Additive exPlanations; PhGA = physician global assessment; AJC = active joint count; CRP = C-reactive protein; (Y/N) = Yes/No. Uni-bilat = unilateral–bilateral; ESR = erythrocyte sedimentation rate; R = right; L = left; MTX = methotrexate; Num of Tx = number of treatments, ANA = antinuclear antibody; RF = rheumatoid factor.

Feature importance is crucial for post hoc explainability of complex models like Random Forests and for identifying the most relevant clinical features [29]. The relevance of all clinical features was assessed in each training and testing set, and they were ranked in decreasing order of importance, as depicted in Figure 2 and Figure 3. In forecasting the ID status at 24 months, the most relevant features were found to be PhGA and AJC, as illustrated in Figure 2. Acute phase reactants, such as erythrocyte sedimentation rate and C-reactive protein, and the involvement of knee and ankle joints, also exhibited some importance, whereas the remaining clinical features proved less relevant.

PhGA and AJC consistently showed significantly greater importance compared to other features across the T0-T6-T24 (Figure 2a), T0-T6-T12-T24 (Figure 2b), and T0-T6-T12-T18-T24 datasets (Figure 2c). This finding underscores their robust capability to forecast the achievement of the ID status at 24 months.

The role of features in forecasting the ID status at 24 months was further investigated using SHAP (SHapley Additive exPlanations) values in the testing sets, which facilitates a global explainability analysis (Figure 3). Across the T0-T6-T24 (Figure 3a), T0-T6-T12-T24 (Figure 3b), and T0-T6-T12-T18-T24 (Figure 3c) datasets, PhGA and AJC were confirmed as the features with the highest impact. The analysis indicated that lower values of PhGA and AJC were closely related to the attainment of ID at 24 months. The T0-T6 dataset is not included in Figure 3 because for this time interval no feature was found to be important in the training set or had an impact greater that zero in the testing dataset.

## 4. Discussion

The use of ML techniques for longitudinal data analysis can reveal hidden patterns that may be difficult to detect in cross-sectional studies. This is because historical patient data can be utilized to build the model. Forecasting involves fitting a model to historical, time-stamped data in order to predict future values of a single variable by leveraging the sequential nature of the data. Traditional statistical approaches, such as Moving Average, Exponential Smoothing, or AutoRegressive Integrated Moving Average [30], have been successfully applied in the healthcare domain [31]. However, these methods were not suitable for our longitudinal study because our model needed to include multiple features, along with an output variable for a large set of patients. In contrast, multivariate time series forecasting methods, such as MLforecast, allowed us to include both binary reference variables and multiple input features for a set of patients longitudinally. ML forecast provides publicly available open source libraries that simplify AI programming and could be coupled with Random Forests or other ML models such as penalized logistic regression, KNN, or SVM. However, Random Forests was the model that obtained the best performance across datasets and was selected for further analyses. Despite Random Forests being a black-box model with limited interpretability, it is a well-known ML technique suitable for prediction and feature importance analysis [27]. For these reasons, we employed MLforecast in our analysis. We used real or estimated values as exogenous features for predicting ID at 2 years after the first observation.

Among the different combinations of time points examined, the forecasting model developed for the T0-T6-T12-T24 dataset demonstrated the best effectiveness in predicting the ID status at 24 months. This finding indicates that the 12-month time frame is the optimal interval for evaluating the capability of clinical outcome measures to forecast the achievement of ID.

Our analysis revealed that the PhGA was consistently the best predictor of achievement of ID at 24 months. This finding indicates that the subjective and objective quantitative estimations of the overall level of disease activity by the caring physician at the time of the visit plays a major role in forecasting the future achievement of ID, and that its decrease over time ensures that the patient is in the best trajectory to reach the state of ID. It also underscores the fundamental importance of regularly performing and recording the PhGA during clinical follow-up of patients with JIA.

The PhGA is a key outcome measure for evaluating disease activity in JIA. It is a complex construct that integrates the information obtained from a clinical history with the findings of a clinical assessment. The PhGA has been found to possess strong responsiveness to clinically important change [32] and to be a valid and reliable indicator of overall JIA activity across all stages of the illness [33]. PhGA scores at disease onset predicted disease trajectory after five years [34]. Owing to its good measurement properties, the PhGA has been incorporated into the main composite endpoints for JIA [16,35,36]. However, previous analyses have highlighted a frequent heterogeneity in rating the PhGA across physicians [37,38]. A recent multinational effort has developed recommendations for scoring the PhGA in children with JIA, aiming to enhance the reliability and comparability of disease activity measurement for clinical care and international clinical trials [39].

The second predictive factor in order of importance was the AJC, which is another physician-centered outcome measure. Backström and co-workers found that the most important factors affecting the PhGA score in patients with non-systemic JIA were the swollen and tender joint count [40]. Guidelines for performing a standardized joint assessment in JIA have been provided [13,14]. However, concern was raised by Alongi et al., who found that many pediatric rheumatologists did not mark a score of 0 for patients who lacked active joints. The presence of pain in those joints not meeting the definition of active arthritis used in JIA was the main determinant of this phenomenon [41]. This observation is consistent with the above considerations about the usefulness of practical guidance for scoring the PhGA in JIA. The number of patients with sJIA included in our cohort was relatively low; we acknowledge that the use of active joint count as a predictive variable may still be relevant for this subgroup. Nevertheless, sJIA can occasionally present with predominant polyarticular joint involvement, especially in patients with a chronic disease course. In such cases, the active joint count may serve as a relevant and clinically meaningful parameter for monitoring disease activity and guiding therapeutic decisions, similarly to other JIA categories. However, due to the distinct pathophysiology and clinical heterogeneity of sJIA, further studies specifically designed to validate predictive models in this subgroup are warranted.

Our study is not without limitations. Patient data were collected through the retrospective review of clinical charts. A retrospective analysis is subject to missing and possibly erroneous data. We did not investigate whether children who were taken off DMARDs within the first year were more likely to experience active disease or relapse. We could not investigate the role of novel biomarkers or imaging methods, especially ultrasound, either in predicting ID or in assessing the state of ID at 24 months. It has been argued that these methodologies may establish disease remission more reliably than clinical assessment [42,43]. The methodology used in our study did not enable us to assess the relationship between variables and the study outcome by calculating numerical thresholds but only allowed us to assess the relative importance of each variable in forecasting ID. Given the low number of patients with sJIA, we could not conclude that our prediction model is as accurate for patients with this JIA category as for those with non-systemic forms. Due to the lack of data in a sizeable number of patients and visits, we could not incorporate the parent/patient assessment of well-being and pain intensity among the study predictors. Therefore, we should recognize that our results do not reflect the potential predictive role of the parent/patient perception of disease status and course. We should finally acknowledge that other items not captured in this study, such as intolerance of medication, might have influenced achievement of ID. The main strength of our study lies in demonstrating, through ML analysis, the capability of our AI model to predict the state of ID at 24 months using empirical exogenous features defined with data obtained from 0 to 12 months. This finding, together with the observed lack of any predictive value of the baseline data, underscores the potential superiority of assessing longitudinal data from multiple follow-up visits, over the simple evaluation of data collected at disease onset, in forecasting long-term achievement of ID.

In conclusion, our study is the first to investigate the role of ML techniques in the prediction of ID in patients with JIA. Through the analysis of longitudinal models built with data collected at subsequent longitudinal visits, we found that the PhGA and the AJC over the first 12 months were the strongest predictors of achieving ID at 24 months. This finding underscores the fundamental importance of regularly measuring the level of disease activity in daily clinical practice using standardized and well-validated outcome measures. These preliminary results highlight the potential of ML techniques in predicting ID in JIA patients; however, further multicenter studies with larger cohorts are necessary to confirm and generalize these results. Future applications of AI methods should integrate clinical information with genetic, multiomic, and imaging data in order to develop robust predictive clinical algorithms that help to optimize the management of JIA through a more precise and personalized therapeutic approach.

## Figures and Tables

**Table 1:** Baseline features of the 414 JIA patients *.

	N (%) or Median (IQR)	N with Available Information
Demographic features		
Females	308 (74.4)	414
Median (IQR) age at disease onset (years)	3.1 (1.8–7.0)	414
Median (IQR) age at study entry (years)	3.2 (2.0–7.1)	414
Median (IQR) disease duration at study entry (months)	1.9 (1–3.4)	414
Functional phenotype		
Systemic arthritis	29 (7)	29
Polyarthritis ^a^	211 (51)	211
Oligoarthritis	158 (38.2)	158
Other arthritis ^b^	16 (3.9)	16
Antinuclear antibody-positive	268 (65.1)	414
Uveitis	15 (3.6)	414
Clinical outcome measures		
Median (IQR) physician’s global assessment	4 (3–6)	414
Active joint count	2 (1–4)	414
Acute phase reactants		
Median (IQR) erythrocyte sedimentation rate	33 (17–51)	356
Median (IQR) C-reactive protein	0.8 (0.5–2.3)	359
Joints involved		
Temporomandibular	16 (3.9)	414
Cervical spine	12 (2.9)	414
Shoulder	17 (4.1)	414
Elbow	59 (14.3)	414
Wrist	77 (18.6)	414
Small hand joints	116 (28)	414
Sacroiliac	2 (0.5)	414
Hip	23 (5.6)	414
Knee	319 (77.1)	414
Ankle	187 (45.2)	414
Small foot joints	81 (19.6)	414

* Data are the number (percentage) unless otherwise indicated. IQR, interquartile range. ^a^ 11 rheumatoid factor-positive; ^b^ 6 enthesitis-related arthritis, 5 psoriatic arthritis, and 5 undifferentiated arthritis.

**Table 2:** Cumulative frequency of the medications administered during the 24-month follow-up period *.

Treatments	N (%)
NSAIDs	346 (83.6)
Intra-articular glucocorticoids	291 (70.3)
Systemic glucocorticoids	113 (27.3)
Methotrexate	273 (65.9)
Sulfasalazine	5 (1.2)
Cyclosporine	3 (0.7)
Etanercept	58 (14)
Adalimumab	18 (4.3)
Infliximab	1 (0.2)
Tocilizumab	3 (0.7)
Abatacept	0 (0)
Anakinra	14 (3.4)
Canakinumab	5 (1.2)
Tofacitinib	0 (0)
Baricitinib	1 (0.2)

* Data are the number (percentage). NSAIDs, non-steroidal anti-inflammatory drugs.

**Table 3:** Forecasting performance of Random Forests coupled with the MLforecast model based on all clinical features and selected time points.

Dataset	MCC Training Set	MCC Testing Set
T0-T6-T12-T18-T24	0.70	0.42
T0-T6-T12-T24	0.68	0.65
T0-T6-T24	0.57	0.50
T0-T24	0.0	0.0

MCC = Matthews Correlation Coefficient. 0 ≤ MCC ≤ 0.19 (Very low), 0.2 ≤ MCC ≤ 0.39 (low), 0.4 ≤ MCC ≤ 0.59 (Moderate), 0.6 ≤ MCC ≤ 0.79 (high), and 0.8 ≤ MCC ≤ 1.0 (very high).

## Data Availability

The anonymized clinical data used in this manuscript have been deposited on the FigShare platform and are available at DOI: 10.6084/m9.figshare.28429931.

## Supplementary Materials

The following supporting information can be downloaded at https://www.mdpi.com/article/10.3390/children12060741/s1, Table S1. Predictor variables assessed in the analyses; Table S2. Patient features by time points for each dataset (T0-T24, T0-T6-T24, T0-T6-T12-T24, and T0-T6-T12-T18-T24); Table S3: Forecasting MCC of additional AI algorithms coupled with the MLforecast model based on all clinical features and selected time points.

## Abbreviations

The following abbreviations are used in this manuscript:

AI	Artificial Intelligence
AJC	Active Joint Count
ANA	Antinuclear Antibody
BOCF	Baseline Observation Carried Forward
CRP	C-Reactive Protein
ESR	Erythrocyte Sedimentation Rate
ID	Inactive Disease
ILAR	International League of Associations for Rheumatology
JIA	Juvenile Idiopathic Arthritis
LOCF	Last Observation Carried Forward
MCC	Matthews Correlation Coefficient
ML	Machine Learning
PhGA	Physician Global Assessment
RF	Rheumatoid Factor
SHAP	SHapley Additive exPlanations
