# Supplementary material for: Forecasting Achievement of Inactive Disease in Juvenile Idiopathic Arthritis with Artificial Intelligence
Source: Children (Basel). 2025 Jun 7;12(6):741. doi: 10.3390/children12060741 (PMC12191878; doi:10.3390/children12060741)
Supplement: Supplementary file 1 [file children-12-00741-s001.zip › Supplementary Table S1.pdf]

Supplementary Table 1 Predictor variables assessed in the analyses

General features

- Follow up visit
- Sex
- Age at disease onset
- Functional phenotype
- Antinuclear antibody
- Rheumatoid factor
- HLA B27
- Physician global assessment
- Active joint count
- ESR
- C-Reactive protein
- Systemic symptoms
- Uveitis

Involved joints

- Right TMJ
- Left TMJ
- TMJ (U/B)
- TMJ (Y/N)
- Cervical spine
- R shoulder
- L shoulder
- Shoulder (U/B)
- Shoulder (Y/N)
- R elbow
- L elbow
- Elbow (U/B)
- Elbow (Y/N)
- R wrist
- L wrist
- Wrist (U/B)
- Wrist (YN)
- R small hand joints
- L small hand joints
- Small hand joints (U/B)
- Small hand joints (Y/N)
- R sacroiliac joint
- L sacroiliac joint
- Sacroiliac joint (U/B)
- Sacroiliac joint (Y/N)
- R hip
- L hip
- Hip (U/B)
- Hip (Y/N)
- R knee
- L knee
- Knee (U/B)
- Knee (Y/N)
- R ankle
- L ankle
- Ankle (U/B)
- Ankle (Y/N)
- R small foot joints
- L small foot joints

Small foot joints (U/B)	Sulfasalazine	Baricitinib
Small foot joints (Y/N)	Cyclosporine	Infliximab
**Medications received**	Etanercept	No. of medications
NSAIDs	Adalimumab	
Intraarticular GCs	Tocilizumab	
Systemic GCs	Anakinra	
Methotrexate	Canakinumab	

ESR = Erythrocyte sedimentation rate; R = right; L = left; TMJ = temporomandibular joint; U/B = unilateral or bilateral; Y/N = yes/no; NSAIDs = nonsteroidal anti-inflammatory drugs; GCs = glucocorticoids
