# Supplementary material for: Forecasting Achievement of Inactive Disease in Juvenile Idiopathic Arthritis with Artificial Intelligence
Source: Children (Basel). 2025 Jun 7;12(6):741. doi: 10.3390/children12060741 (PMC12191878; doi:10.3390/children12060741)
Supplement: Supplementary file 1 [file children-12-00741-s001.zip › Supplementary Table S2.pdf]

Supplementary Table 2 Patient features by time points for each dataset (T_0-T_{24}, T_0-T_6-T_{24}, T_0-T_6-T_{12}-T_{24}, and T_0-T_6-T_{12}-T_{18}-T_{24}). Proportion and relative percentage (brackets) of patients were reported for categorical features. Median and interqualtile range (brackets) were reported for numerical features

	T0-T6-T12-T18-T24					T0-T6-T12-T24				T0-T6-T24			T0-T24	
**Features**	**T0**	**T6**	**T12**	**T18**	**T24**	**T0**	**T6**	**T12**	**T24**	**T0**	**T6**	**T24**	**T0**	**T24**
Sex														
Male	41 (20.4)	41 (20.4)	41 (20.4)	41 (20.4)	41 (20.4)	70 (23.2)	70 (23.2)	70 (23.2)	70 (23.2)	79 (23.3)	79 (23.3)	79 (23.3)	79 (23.3)	79 (23.)
Female	160 (79.6)	160 (79.6)	160 (79.6)	160 (79.6)	160 (79.6)	232 (76.8)	232 (76.8)	232 (76.8)	232 (76.8)	260 (76.7)	260 (76.7)	260 (76.7)	260 (76.7)	260 (76.7)
Functional phenotypes														
Systemic	9 (4.5)	9 (4.5)	9 (4.5)	9 (4.5)	9 (4.5)	14 (4.6)	14 (4.6)	14 (4.6)	14 (4.6)	17 (5.0)	17 (5.0)	17 (5.0)	17 (5.0)	17 (5.0)
Oligoarticular Persistent	103 (51.2)	103 (51.2)	103 (51.2)	103 (51.2)	103 (51.2)	157 (52)	157 (52)	157 (52)	157 (52)	173 (51)	173 (51)	173 (51)	173 (51)	173 (51)
Polyarticular	80 (39.8)	80 (39.8)	80 (39.8)	80 (39.8)	80 (39.8)	119 (39.4)	119 (39.4)	119 (39.4)	119 (39.4)	135 (39.8)	135 (39.8)	135 (39.8)	135 (39.8)	135 (39.8)
Other	9 (4.5)	9 (4.5)	9 (4.5)	9 (4.5)	9 (4.5)	12 (4.0)	12 (4.0)	12 (4.0)	12 (4.0)	14 (4.1)	14 (4.1)	14 (4.1)	14 (4.1)	14 (4.1)
ANA														
Positive	141 (70.1)	141 (70.1)	141 (70.1)	141 (70.1)	141 (70.1)	205 (67.9)	205 (67.9)	205 (67.9)	205 (67.9)	227 (67.2)	227 (67.2)	227 (67.2)	227 (67.2)	227 (67.2)
Negative	59 (29.4)	59 (29.4)	59 (29.4)	59 (29.4)	59 (29.4)	96 (31.8)	96 (31.8)	96 (31.8)	96 (31.8)	111 (32.7)	111 (32.7)	111 (32.7)	111 (32.7)	111 (32.7)
Missing	1 (0.5)	1 (0.5)	1(0.5)	1 (0.5)	1 (0.5)	1 (0.3)	1(0.3)	1(0.3)	1(0.3)	1 (0.3)	1(0.3)	1(0.3)	1(0.3)	1(0.3)
RF														
Positive	7 (3.5)	7 (3.5)	7 (3.5)	7 (3.5)	7 (3.5)	11 (3.6)	11 (3.6)	11 (3.6)	11 (3.6)	12 (3.5)	12 (3.5)	12 (3.5)	12 (3.5)	12 (3.5)
Negative	193 (96.0)	193 (96.0)	193 (96.0)	193 (96.0)	193 (96.0)	290 (96.0)	290 (96.0)	290 (96.0)	290 (96.0)	326 (96.2)	326 (96.2)	326 (96.2)	326 (96.2)	326 (96.2)
Missing	1 (0.5)	1 (0.5)	1(0.5)	1 (0.5)	1 (0.5)	1 (0.3)	1(0.3)	1(0.3)	1(0.3)	1(0.3)	1(0.3)	1(0.3)	1(0.3)	1(0.3)

	T0-T6-T12-T18-T24					T0-T6-T12-T24				T0-T6-T24			T0-T24	
**Features**	**T0**	**T6**	**T12**	**T18**	**T24**	**T0**	**T6**	**T12**	**T24**	**T0**	**T6**	**T24**	**T0**	**T24**
HLA B27														
Positive	4 (2)	4 (2)	4 (2)	4 (2)	4 (2)	6 (2)	6 (2)	6 (2)	6 (2)	7 (2.1)	7 (2.1)	7 (2.1)	7 (2.1)	7 (2.1)
Negative	196 (97.5)	196 (97.5)	196 (97.5)	196 (97.5)	196 (97.5)	295 (97.7)	295 (97.7)	295 (97.7)	295 (97.7)	331 (97.6)	331 (97.6)	331 (97.6)	331 (97.6)	331 (97.6)
Missing	1 (0.5)	1 (0.5)	1 0.5)	1 (0.5)	1 (0.5)	1 (0.3)	1(0.3)	1(0.3)	1(0.3)	1 (0.3)	1(0.3)	1(0.3)	1(0.3)	1(0.3)
Age at onset	2.7 (1.8-5.8)	2.7 (1.8-5.8)	2.7 (1.8-5.8)	2.7 (1.8-5.8)	2.7 (1.8-5.8)	2.8 (1.8-6.7)	2.8 (1.8-6.7)	2.8 (1.8-6.7)	2.8 (1.8-6.7)	2.8 (1.8-6.8)	2.8 (1.8-6.8)	2.8 (1.8-6.8)	2.8 (1.8-6.8)	2.8 (1.8-6.8)
PhGA	4 (3-6)	1 (0-3)	0 (0-2)	0 (0-1)	0 (0-1)	4 (3-6)	1 (0-3)	0 (0-2)	0 (0-1)	4 (3-6)	1 (0-3)	0 (0-1)	4 (3-6)	0 (0-1)
AJC	2 (1-4)	1 (0-2)	0 (0-2)	0 (0-1)	0 (0-1)	2 (1-4)	1 (0-2)	0 (0-1)	0 (0-1)	2 (1-4)	1 (0-2)	0 (0-1)	2 (1-4)	0 (0-1)
ESR	33 (16 - 50)	13 (8-26)	11 (7-24)	10 (7-18)	9 (6-13.8)	32.5 (17-50.3)	12 (8-25.2)	10 (7-21)	9 (6-14)	32 (17-50)	12 (8-26)	9 (6-14)	32 (17-50)	9 (6-14)
Missing	24 (11.9)	56 (27.9)	61 (30.3)	48 (23.9)	59 (29.4)	42 (13.9)	94 (31.3)	97 (32.1)	89 (29.5)	47 (13.9)	108 (31.9)	105 (31)	47 (13.9)	105 (31)
CRP	0.79 (0.45-2.24)	0.45 (0.45-0.56)	0.45 (0-0.46)	0.45 (0..04-0.46)	0.45 (0..04-0.46)	0.83 (0.45-2.24)	0.45 (0.4-0-5)	0.45 (0-0.46)	0.45 (0-0.46)	0.8 (0.45-2.1)	0.45 (0-0.45)	0.45 (0-0.45)	0.8 (0.45-2.1)	0.45 (0-0.45)
Missing	26 (12.9)	53 (26.4)	57 (28.4)	51 (25.4)	57 (28.4)	41 (13.6)	89 (28.8)	94 (31.3)	89 (29.5)	44 (13)	99 (29.2)	105 (31)	44 (13)	105 (31)
Systemic symptoms														
Yes	12 (6.0)	0 (0)	1 (0.5)	0 (0)	2 (1)	20 (6.6)	0 (0)	2 (0.7)	2 (0.7)	23 (6.8)	0 (0)	2 (0.6)	23 (6.8)	2 (0.6)
No	189 (94)	201 (100)	200 (99.5)	201 (100)	199 (99)	282 (93.4)	302 (100)	300 (99.3)	300 (99.3)	316 (93.2)	339 (100)	337 (99.4)	316 (93.2)	337 (99.4)

Features		T0-T6-T12-T18-T24					T0-T6-T12-T24				T0-T6-T24			T0-T24	
		T0	T6	T12	T18	T24	T0	T6	T12	T24	T0	T6	T24	T0	T24
Uveitis															
	Yes	6 (3)	6 (3.0)	9 (4.5)	11 (5.5)	9 (4.5)	10 (3.3)	10 (3.2)	16 (5.3)	13 (4.3)	10 (2.9)	11 (3.2)	13 (3.8)	10 (2.9)	13 (3.8)
	No	195 (97.0)	195 (97)	192 (95.5)	190 (94.5)	192 (95.5)	296 (96.7)	292 (96.7)	286 (94.7)	289 (95.7)	329 (97.1)	328 (96.8)	326 (96.2)	329 (97.1)	326 (96.2)
R TMJ															
	Yes	3 (1.5)	2 (1)	4 (2.0)	4 (2.0)	1 (0.5)	6 (2)	5 (1.7)	5 (1.7)	5 (1.7)	8 (2.4)	6 (1.8)	6 (1.8)	8 (2.4)	6 (1.8)
	No	198 (98.5)	199 (99)	197 (98.0)	197 (98.0)	200 (99.5)	296 (98)	297 (98.3)	297 (98.3)	297 (98.3)	331 (97.6)	333 (98.2)	333 (98.2)	331 (97.6)	333 (98.2)
L TMJ															
	Yes	5 (2.5)	1 (0.5)	5 (2.5)	4 (2.0)	0 (0)	9 (3)	6 (2.0)	6 (2.0)	4 (1.3)	11 (3.2)	6 (1.8)	5 (1.5)	11 (3.2)	5 (1.5)
	No	196 (97.5)	200 (99.5)	196 (97.5)	197 (98.0)	201 (100)	293 (97.0)	296 (98)	296 (98)	298 (98.7)	328 (96.8)	333 (98.2)	334 (98.5)	328 (96.8)	334 (98.5)
TMJ uni-bilat															
	No	195 (97.0)	199 (99)	194 (96.5)	197 (98.0)	200 (99.5)	291 (96.4)	294 (97.4)	293 (97.0)	295 (97.7)	325 (95.9)	331 (97.6)	331 (97.6)	325 (95.9)	331 (97.6)
	Unilateral	4 (2)	1 (0.5)	5 (2.5)	4 (2.0)	1 (0.5)	7 (2.3)	5 (1.7)	7 (2.3)	5 (1.7)	9 (2.7)	5 (1.5)	5 (1.5)	9 (2.7)	5 (1.5)
	Bilateral	2 (1)	1 (0.5)	2 (1)	0 (0)	0 (0)	4 (1.3)	3(1.0)	2(0.7)	2(0.7)	5 (1.5)	3(0.9)	3(0.9)	5(1.5)	3(0.9)
TMJ involvement (Y/N)															
	Yes	6 (3)	2 (1)	7 (3.5)	4 (2.0)	1 (0.5)	11 (3.6)	8 (2.6)	9 (3)	7 (2.3)	14 (4.1)	8 (2.4)	8 (2.4)	14 (4.1)	8 (2.4)
	No	195 (97.0)	199 (99)	194 (96.5)	197 (98.0)	200 (99.5)	291 (96.4)	294 (97.4)	293 (97.0)	295 (97.7)	325 (95.9)	331 (97.6)	331 (97.6)	325 (95.9)	331 (97.6)
Cervical column															
	Yes	5 (2.5)	2 (1)	4 (2)	4 (2.0)	3 (1.5)	9 (3.0)	6 (2)	4 (1.3)	4 (1.3)	10 (2)	6 (1.8)	4 (1.1)	10 (2.9)	4 (1.1)

	T0-T6-T12-T18-T24					T0-T6-T12-T24				T0-T6-T24			T0-T24	
**Features**	**T0**	**T6**	**T12**	**T18**	**T24**	**T0**	**T6**	**T12**	**T24**	**T0**	**T6**	**T24**	**T0**	**T24**
No	196 (97.5)	199 (99)	197 (98)	197 (98.0)	198 (98.5)	293 (97)	296 (98)	298 (98.7)	298 (98.7)	329 (97.1)	333 (98.2)	335 (98.8)	329 (97.1)	335 (98.8)
R shoulder														
Yes	8 (4)	2 (1)	3 (1.5)	1 (0.5)	3 (1.5)	13 (4.3)	2 (0.7)	3 (1)	3 (1)	14 (4.1)	3 (0.9)	3 (0.9)	14 (4.1)	3 (0.9)
No	193 (96.0)	199 (99)	198 (98.5)	200 (99.5)	198 (98.5)	289 (95.7)	300 (99.3)	299 (99)	299 (99)	325 (95.9)	336 (99.1)	336 (99.1)	325 (95.9)	336 (99.1)
L shoulder														
Yes	4 (2)	1 (0.5)	3 (1.5)	1 (0.5)	1 (0.5)	8 (2.6)	2 (0.7)	3 (1)	1 (0.3)	8 (2.4)	2 (0.6)	2 (0.6)	8 (2.4)	2 (0.6)
No	197 (98.0)	200 (99.5)	198 (98.5)	200 (99.5)	200 (99.5)	293 (97)	300 (99.3)	299 (99)	300 (99.3)	330 (97.3)	337 (99.4)	336 (99.1)	330 (97.3)	336 (99.1)
missing	0 (0)	0 (0)	0 (0)	0 (0)	0 (0)	1(0.3)	0 (0)	0 (0)	1(0.3)	1 (0.3)	0 (0)	1 (0.3)	1 (0.3)	1 (0.3)
Shoulder uni-bilat														
No	192 (95.5)	198 (98.5)	196 (97.5)	200 (99.5)	198 (98.5)	288 (95.4)	298 (98.7)	297 (98.3)	299 (99)	324 (95.6)	334 (98.5)	335 (98.9)	324 (95.6)	335 (98.9)
Unilateral	6 (3)	3 (1.5)	4 (2)	0 (0)	2 (1)	7 (2.3)	4 (1.3)	4 (1.3)	2 (0.7)	8 (2.4)	5 (1.5)	3 (0.9)	8 (2.4)	3 (0.9)
Bilateral	3 (1.5)	0 (0)	1 (0.5)	1 (0.5)	1 (0.5)	7 (2.3)	0 (0)	1 (0.3)	1 (0.3)	7 (2.1)	0 (0)	1 (0.3)	7 (2.1)	1 (0.3)
Shoulder involvement (Y/N)														
Yes	9 (4.5)	3 (1.5)	5 (2.5)	1 (0.5)	3 (1.5)	14 (4.6)	4 (1.3)	5 (1.7)	3 (1)	15 (4.4)	1 (5)	4 (1.2)	15 (4.4)	4 (1.2)
No	192 (95.5)	198 (98.5)	196 (97.5)	200 (99.5)	198 (98.5)	288 (95.4)	298 (98.7)	297 (98.3)	299 (99)	324 (95.6)	334 (98.5)	355 (98.9)	324 (95.6)	355 (98.9)

	T0-T6-T12-T18-T24					T0-T6-T12-T24				T0-T6-T24			T0-T24	
**Features**	**T0**	**T6**	**T12**	**T18**	**T24**	**T0**	**T6**	**T12**	**T24**	**T0**	**T6**	**T24**	**T0**	**T24**
R elbow														
Yes	16 (8.0)	11 (5.5)	5 (2.5)	1 (0.5)	2 (1)	25 (8.3)	15 (5)	8 (2.6)	2 (0.7)	33 (9.7)	20 (5.9)	2 (0.6)	33 (9.7)	2 (0.6)
No	185 (92)	190 (94.5)	196 (97.5)	200 (99.5)	199 (99)	277 (91.7)	287 (95)	294 (97.4)	300 (99.3)	306 (90.3)	319 (94.1)	337 (99.4)	306 (90.3)	337 (99.4)
L elbow														
Yes	12 (6.0)	12 (6)	7 (3.5)	5 (2.5)	3 (1.5)	23 (7.6)	14 (4.6)	11 (3.6)	3 (1)	29 (8.6)	14 (4.1)	3 (0.9)	29 (8.6)	3 (0.9)
No	189 (94)	189 (94.0)	194 (96.5)	196 (97.5)	198 (98.5)	279 (92.4)	288 (95.4)	291 (96.4)	299 (99)	310 (91.4)	325 (95.9)	336 (99.1)	310 (91.4)	336 (99.1)
Elbow uni-bilat														
No	179 (89.1)	180 (89.6)	191 (95)	196 (97.5)	197 (98)	264 (87.4)	277 (91.7)	286 (94.7)	298 (98.7)	292 (86.1)	309 (91.2)	335 (98.8)	292 (86.1)	335 (98.8)
Unilateral	16 (8.0)	19 (9.5)	8 (4)	4 (2)	3 (1.5)	28 (9.3)	21 (7)	13 (4.1)	3 (1)	32 (9.4)	26 (7.7)	3 (0.9)	32 (9.4)	3 (0.9)
Bilateral	6 (3.0)	2 (1.0)	2 (1)	1 (0.5)	1 (0.5)	10 (3.3)	4 (1.3)	3 (1)	1 (0.3)	15 (4.4)	4 (1.2)	1 (0.3)	15 (4.4)	1 (0.3)
Elbow involvement (Y/N)														
Yes	22 (10.9)	21 (10.4)	10 (5)	5 (2.5)	4 (2)	38 (12.6)	25 (8.3)	16 (5.3)	4 (1.3)	47 (13.9)	30 (8.8)	4 (1.2)	47 (13.9)	4 (1.2)
No	179 (89.1)	180 (89.6)	191 (95)	196 (97.5)	197 (98)	264 (87.4)	277 (91.7)	286 (94.7)	298 (98.7)	292 (86.1)	309 (91.2)	335 (98.9)	292 (86.1)	335 (98.9)
R wrist														
Yes	27 (13.4)	15 (7.5)	8 (4.0)	4 (2)	9 (4.5)	39 (12.9)	21 (7)	11 (3.6)	13 (4.3)	49 (14.5)	25 (7.4)	15 (4.4)	49 (14.5)	15 (4.4)
No	174 (86.6)	186 (92.5)	193 (96)	197 (98)	192 (95.5)	263 (87.1)	281 (93)	291 (96.4)	289 (95.7)	290 (85.5)	314 (92.6)	324 (95.6)	290 (85.5)	324 (95.6)

	T0-T6-T12-T18-T24					T0-T6-T12-T24				T0-T6-T24			T0-T24	
**Features**	**T0**	**T6**	**T12**	**T18**	**T24**	**T0**	**T6**	**T12**	**T24**	**T0**	**T6**	**T24**	**T0**	**T24**
L wrist														
Yes	24 (11.9)	9 (4.5)	7 (3.5)	4 (2)	5 (2.5)	34 (11.3)	14 (4.6)	9 (3)	6 (2)	42 (12.4)	18 (5.3)	7 (2.1)	42 (12.4)	7 (2.1)
No	177 (88.1)	192 (95.5)	194 (96.5)	197 (98)	196 (97.5)	268 (88.7)	288 (95.4)	293 (97)	296 (98)	297 (87.6)	321 (94.7)	332 (97.9)	297 (87.6)	332 (97.9)
Wrist uni-bilat														
No	166 (82.6)	182 (90.5)	191 (95)	195 (97)	191 (95)	252 (83.4)	275 (91.1)	288 (95.4)	288 (95.4)	278 (82)	307 (90.6)	323 (95.3)	278 (82)	323 (95.3)
Unilateral	19 (9.5)	14 (7)	5 (2.5)	4 (2)	6 (3)	27 (8.9)	19 (6.3)	8 (2.6)	9 (3)	31 (9.1)	21 (6.2)	10 (2.9)	31 (9.1)	10 (2.9)
Bilateral	16 (8.0)	5 (2.5)	5 (2.5)	2 (1)	4 (2)	23 (7.6)	8 (2.6)	6 (2)	5 (1.7)	30 (8.8)	11 (3.2)	6 (1.8)	30 (8.8)	6 (1.8)
Wrist involvement (Y/N)														
Yes	35 (17.4)	19 (9.5)	10 (5.0)	6 (3)	10 (5)	50 (16.6)	27 (8.9)	14 (4.6)	14 (4.6)	61 (18)	32 (9.4)	16 (4.7)	61 (18)	16 (4.7)
No	166 (82.6)	182 (90.5)	191 (95)	195 (97)	191 (95)	252 (83.4)	275(91.1)	288 (95.4)	288 (95.4)	278 (82)	307 (90.6)	323 (95.3)	278 (82)	323 (95.3)
R small hand j.														
Yes	45 (22.4)	30 (14.9)	27 (13.4)	13 (6.5)	17 (8.5)	66 (21.9)	45 (14.9)	33 (10.9)	22 (7.3)	77 (22.7)	50 (14.7)	24 (7.1)	77 (22.7)	24 (7.1)
No	156 (77.6)	171 (85.1)	174 (86.6)	188 (93.5)	184 (91.5)	236 (78.1)	257 (85.1)	269 (89.1)	280 (92.7)	262 (77.3)	289 (85.3)	315 (92.9)	262 (77.3)	315 (92.9)
L small hand j.														
Yes	40 (19.9)	30 (14.9)	27 (13.4)	15 (7.5)	15 (7.5)	60 (19.9)	39 (12.9)	32 (10.6)	18 (6)	69 (20.4)	45 (13.3)	21 (6.2)	69 (20.4)	21 (6.2)
No	161 (80.1)	171 (85.1)	174 (86.6)	186 (92.5)	186 (92.5)	242 (80.1)	263 (87.1)	270 (89.4)	284 (94)	270 (79.6)	294 (86.7)	318 (93.8)	270 (79.6)	318 (93.8)

Features	T0-T6-T12-T18-T24 T0	T6	T12	T18	T24	T0-T6-T12-T24 T0	T6	T12	T24	T0-T6-T24 T0	T6	T24	T0-T24 T0	T24
Small hand j. uni-bilat														
No	144 (71.6)	159 (79.1)	169 (84.1)	183 (91)	180 (89.6)	219 (72.5)	244 (80.8)	261 (86.4)	275 (91.1)	243 (71.7)	274 (80.8)	309 (91.2)	243 (71.7)	309 (91.2)
Unilateral	29 (14.4)	24 (11.9)	10 (5)	8 (4)	10 (5)	43 (14.2)	32 (10.6)	17 (5.6)	14 (4.6)	46 (13.6)	35 (10.3)	15 (4.4)	46 (13.6)	15 (4.4)
Bilateral	28 (13.9)	18 (9.0)	22 (10.9)	10 (5)	11 (5.5)	40 (13.2)	26 (8.6)	24 (7.9)	13 (4.3)	50 (14.7)	30 (8.8)	15 (4.4)	50 (14.7)	15 (4.4)
Small hand j. involvement (Y/N)														
Yes	57 (28.4)	42 (20.9)	32 (15.9)	18 (9)	21 (10.4)	83 (27.5)	58 (19.2)	41 (13.6)	27 (8.9)	98 (28.3)	65 (19.2)	30 (8.8)	98 (28.3)	30 (8.8)
No	144 (71.6)	159 (79.1)	169 (84.1)	183 (91)	180 (89.6)	219 (72.5)	244 (80.8)	261 (86.4)	275 (91.1)	243 (71.7)	274 (80.8)	309 (91.2)	243 (71.7)	309 (91.2)
R sacroiliac														
Yes	1 (0.5)	1 (0.5)	0 (0)	0 (0)	0 (0)	1 (0.3)	1(0.3)	0 (0)	0 (0)	1 (0.3)	1(0.3)	0 (0)	1(0.3)	0 (0)
No	199 (99)	199 (99)	200 (99.5)	200 (99.5)	200 (99.5)	300 (99.3)	300 (99.3)	301 (99.7)	301 (99.7)	337 (99.4)	337 (99.4)	338 (99.7)	337 (99.4)	338 (99.7)
Missing	1 (0.5)	1 (0.5)	1(0.5)	1 (0.5)	1 (0.5)	1 (0.3)	1(0.3)	1(0.3)	1(0.3)	1 (0.3)	1(0.3)	1(0.3)	1(0.3)	1(0.3)
L sacroiliac														
Yes	1 (0.5)	1 (0.5)	0 (0)	0 (0)	0 (0)	1 (0.3)	1(0.3)	0 (0)	0 (0)	1 (0.3)	1(0.3)	0 (0)	1(0.3)	0 (0)
No	199 (99)	199 (99)	200 (99.5)	200 (99.5)	200 (99.5)	300 (99.3)	300 (99.3)	301 (99.7)	301 (99.7)	337 (99.4)	337 (99.4)	338 (99.7)	337 (99.4)	338 (99.7)
Missing	1 (0.5)	1 (0.5)	1(0.5)	1 (0.5)	1 (0.5)	1 (0.3)	1(0.3)	1(0.3)	1(0.3)	1 (0.3)	1(0.3)	1(0.3)	1(0.3)	1(0.3)

	T0-T6-T12-T18-T24					T0-T6-T12-T24				T0-T6-T24			T0-T24	
**Features**	**T0**	**T6**	**T12**	**T18**	**T24**	**T0**	**T6**	**T12**	**T24**	**T0**	**T6**	**T24**	**T0**	**T24**
Sacroiliac uni-bilat														
No	199 (99)	199 (99)	200 (99.5)	200 (99.5)	200 (99.5)	300 (99.3)	300 (99.3)	301 (99.7)	301 (99.7)	337 (99.4)	337 (99.4)	338 (99.7)	337 (99.4)	338 (99.7)
Unilateral	0 (0)	0 (0)	0 (0)	0 (0)	0 (0)	0 (0)	0 (0)	0 (0)	0 (0)	0 (0)	0 (0)	0 (0)	0 (0)	0 (0)
Bilateral	1 (0.5)	1 (0.5)	0 (0)	0 (0)	0 (0)	1 (0.3)	1(0.3)	0 (0)	0 (0)	1 (0.3)	1(0.3)	0 (0)	1(0.3)	0 (0)
Missing	1 (0.5)	1 (0.5)	1(0.5)	1 (0.5)	1 (0.5)	1 (0.3)	1(0.3)	1(0.3)	1(0.3)	1 (0.3)	1(0.3)	1(0.3)	1(0.3)	1(0.3)
Sacroiliac involvement (Y/N)														
Yes	1 (0.5)	1 (0.5)	0 (0)	0 (0)	0 (0)	1 (0.3)	1 (0.3)	0 (0)	0 (0)	1 (0.3)	1 (0.3)	0 (0)	1 (0.3)	0 (0)
No	199 (99)	199 (99)	200 (99.5)	200 (99.5)	200 (99.5)	300 (99.3)	300 (99.3)	301 (99.7)	301 (99.7)	337 (99.4)	337 (99.4)	338 (99.7)	337 (99.4)	338 (99.7)
Missing	1 (0.5)	1 (0.5)	1(0.5)	1 (0.5)	1 (0.5)	1 (0.3)	1(0.3)	1(0.3)	1(0.3)	1 (0.3)	1(0.3)	1(0.3)	1(0.3)	1(0.3)
R hip														
Yes	9 (4.5)	5 (2.5)	3 (1.5)	4 (2)	3 (1.5)	16 (5.3)	6 (2.0)	4 (1.3)	5 (1.7)	18 (5.3)	7 (2.1)	6 (1.8)	18 (5.3)	6 (1.8)
No	192 (95.5)	196 (97.5)	198 (98.5)	197 (98)	198 (98.5)	286 (94.7)	296 (98)	298 (98.7)	297 (98.3)	321 (94.7)	332 (97.9)	331 (98.2)	321 (94.7)	331 (98.2)
L hip														
Yes	7 (3.5)	4 (2)	2 (1.0)	2 (1.0)	2 (1.0)	16 (4.3)	6 (2.0)	5 (1.7)	4 (1.3)	15 (4.4)	7 (2.1)	5 (1.5)	15 (4.4)	5 (1.5)
No	194 (96.5)	197 (98)	199 (99)	199 (99)	199 (99)	289 (95.7)	296 (98)	297 (98.3)	298 (98.7)	324 (95.6)	332 (97.9)	334 (98.5)	324 (95.6)	334 (98.5)

	T0-T6-T12-T18-T24					T0-T6-T12-T24				T0-T6-T24			T0-T24	
**Features**	**T0**	**T6**	**T12**	**T18**	**T24**	**T0**	**T6**	**T12**	**T24**	**T0**	**T6**	**T24**	**T0**	**T24**
Hip uni-bilat														
No	190 (94.5)	194 (96.5)	197 (98)	197 (98)	198 (98.5)	283 (93.7)	293 (97)	295 (97.7)	297 (98.3)	317 (93.5)	329 (97.1)	333 (98.2)	317 (93.5)	333 (98.2)
Unilateral	6 (3.0)	5 (2.5)	3 (1.5)	2 (1.0)	2 (1.0)	10 (3.3)	6 (2.0)	5 (1.7)	4 (1.3)	11 (3.2)	6 (1.8)	1 (0.3)	11 (3.2)	1 (0.3)
Bilateral	5 (2.5)	2 (1.0)	1 (0.5)	2 (1.0)	1 (0.5)	9 (3.0)	3 (1.0)	2 (0.7)	1 (0.3)	11 (3.2)	4 (1.2)	5 (1.5)	11 (3.2)	5 (1.5)
Hip involvement (Y/N)														
Yes	11 (5.5)	7 (3.5)	4 (2)	4 (2)	3 (1.5)	19 (6.3)	9 (3)	7 (2.3)	5 (1.7)	22 (6.5)	10 (2.9)	6 (1.8)	22 (6.5)	6 (1.8)
No	190 (94.5)	194 (96.5)	197 (98)	197 (98)	198 (98.5)	283 (93.7)	293 (97)	295 (97.7)	297 (98.3)	317 (93.5)	329 (97.1)	333 (98.2)	317 (93.5)	333 (98.2)
R knee														
Yes	107 (53.2)	23 (11.4)	11 (5.5)	12 (6)	13 (6.5)	164 (54.3)	39 (12.9)	25 (8.3)	20 (6.6)	183 (54)	44 (13)	22 (6.5)	183 (54)	22 (6.5)
No	96 (46.8)	178 (88.6)	190 (94.5)	189 (94)	188 (93.5)	138 (45.7)	263 (87.1)	277 (91.7)	282 (93.4)	156 (46.0)	295 (87)	317 (93.5)	156 (46.0)	317 (93.5)
L knee														
Yes	107 (53.2)	24 (11.9)	21 (10.4)	11 (5.5)	16 (8)	159 (52)	34 (11.3)	24 (7.9)	27 (8.9)	181 (53.4)	35 (10.3)	29 (8.6)	181 (53.4)	29 (8.6)
No	93 (46.3)	177 (88.1)	180 (89.6)	190 (94.5)	185 (92)	142 (47)	268 (88.7)	278 (92.1)	275 (91.1)	157 (46.3)	304 (89.7)	310 (91.4)	157 (46.3)	310 (91.4)
Missing	1 (0.5)	0 (0)	0 (0)	0 (0)	0 (0)	1 (0.3)	0 (0)	0 (0)	0 (0)	1 (0.3)	0 (0)	0 (0)	1 (0.3)	0 (0)

	T0-T6-T12-T18-T24					T0-T6-T12-T24				T0-T6-T24			T0-T24	
**Features**	**T0**	**T6**	**T12**	**T18**	**T24**	**T0**	**T6**	**T12**	**T24**	**T0**	**T6**	**T24**	**T0**	**T24**
Knee uni-bilat														
No	46 (22.9)	161 (80.1)	172 (85.6)	181 (90)	174 (86.6)	61 (20.2)	242 (80.1)	257 (85.1)	261 (86.4)	71 (20-9)	274 (80.8)	294 (86.7)	71 (20-9)	294 (86.7)
Unilateral	95 (47.3)	33 (16.4)	26 (12.9)	17 (8.5)	25 (12.4)	158 (52.3)	47 (15.6)	41 (13.6)	35 (11.6)	171 (50.4)	51 (15)	39 (11.5)	171 (50.4)	39 (11.5)
Bilateral	59 (29.4)	7 (3.5)	3 (1.5)	3 (1.5)	2 (1)	82 (27.2)	13 (4.3)	4 (1.3)	6 (2)	96 (28.3)	14 (4.1)	6 (1.8)	96 (28.3)	6 (1.8)
Missing	0 (0)	0 (0)	0 (0)	0 (0)	0 (0)	1 (0.3)	0 (0)	0 (0)	0 (0)	1 (0.3)	0 (0)	0 (0)	1 (0.3)	0 (0)
Knee involvement (Y/N)														
Yes	155 (77.1)	40 (19.9)	29 (14.4)	20 (10)	27 (13.4)	241 (79.8)	60 (19.9)	45 (14.9)	41 (13.6)	268 (79.1)	65 (19.2)	45 (13.3)	268 (79.1)	45 (13.3)
No	46 (22.9)	161 (80.1)	172 (85.6)	181 (90)	174 (86.6)	61 (20.2)	242 (80.1)	257 (85.1)	261 (86.4)	71 (20.9)	274 (80.8)	294 (86.7)	71 (20.9)	294 (86.7)
R ankle														
Yes	62 (30.8)	43 (21.4)	20 (10)	19 (9.5)	19 (9.5)	98 (32.5)	57 (18.9)	25 (8.3)	25 (8.3)	114 (33.6)	66 (19.5)	28 (8.3)	114 (33.6)	28 (8.3)
No	139 (69.2)	158 (78.6)	181 (90)	182 (90.5)	182 (90.5)	204 (67.5)	245 (81.1)	277 (91.7)	277 (91.7)	225 (66.4)	273 (80.5)	311 (91.7)	225 (66.4)	311 (91.7)
L ankle														
Yes	57 (28.4)	45 (22.4)	34 (16.9)	16 (8)	20 (10)	82 (27.2)	61 (20.2)	47 (15.6)	31 (10.3)	92 (27.1)	70 (20.6)	34 (10)	92 (27.1)	34 (10)
No	144 (71.6)	156 (77.6)	167 (83.1)	185 (92)	181 (90)	220 (72.8)	241 (79.8)	255 (84.4)	271 (89.7)	247 (72.9)	269 (79.4)	305 (90)	247 (72.9)	305 (90)

	T0-T6-T12-T18-T24					T0-T6-T12-T24				T0-T6-T24			T0-T24	
**Features**	**T0**	**T6**	**T12**	**T18**	**T24**	**T0**	**T6**	**T12**	**T24**	**T0**	**T6**	**T24**	**T0**	**T24**
Ankle uni-bilat														
No	109 (54.2)	131 (65.2)	155 (77.1)	172 (85.6)	167 (83.1)	165 (54.6)	207 (68.5)	238 (78.8)	255 (84.4)	184 (54.3)	231 (68.1)	286 (84.8)	184 (54.3)	286 (84.8)
Unilateral	65 (32.3)	53 (25.9)	38 (18.9)	23 (11.4)	29 (14.4)	94 (31.1)	72 (23.8)	56 (18.5)	38 (12.6)	104 (30.7)	80 (23.6)	44 (13)	104 (30.7)	44 (13)
Bilateral	27 (13.4)	18 (9.0)	8 (4.0)	6 (3)	5 (2.5)	43 (14.2)	23 (7.6)	8 (2.6)	9 (3)	51 (15)	28 (8.3)	9 (2.7)	51 (15)	9 (2.7)
Ankle involvement (Y/N)														
Yes	92 (45.8)	70 (34.8)	46 (22.9)	29 (14.4)	34 (16.9)	137 (45.4)	95 (31.5)	64 (21.2)	47 (15.6)	155 (45.7)	108 (31.9)	53 (15.6)	155 (45.7)	53 (15.6)
No	109 (54.2)	131 (65.2)	155 (77.1)	172 (85.6)	167 (83.1)	165 (54.6)	207 (68.5)	238 (78.8)	255 (84.4)	184 (54.3)	231 (68.1)	286 (84.8)	184 (54.3)	286 (84.8)
R small foot j														
Yes	30 (14.9)	16 (8.0)	16 (8)	14 (7)	11 (5)	40 (13.2)	29 (9.6)	21 (7)	12 (4)	46 (13.6)	32 (9.4)	12 (3.3)	46 (13.6)	12 (3.3)
No	171 (85.1)	185 (92)	185 (92)	187 (93)	190 (94.5)	262 (86.8)	273 (90.4)	281 (93)	290 (96)	293 (86.4)	307 (90.6)	327 (96.5)	293 (86.4)	327 (96.5)
L small foot j														
Yes	28 (13.9)	16 (8)	11 (5.5)	12 (6)	8 (4)	36 (11.9)	23 (7.6)	18 (6)	11 (3.6)	41 (12.1)	27 (8)	13 (3.8)	41 (12.1)	13 (3.8)
No	173 (86.1)	185 (92)	190 (94.5)	189 (94)	193 (96)	266 (88.1)	279 (92.4)	284 (94)	291 (96.4)	298 (87.9)	312 (92)	326 (96.2)	298 (87.9)	326 (96.2)
Small foot j uni-bilat														
No	157 (78.1)	175 (87)	181 (90.0)	181 (90)	186 (92.5)	244 (80.8)	261 (86.4)	273 (90.4)	284 (94)	273 (80.5)	294 (86.7)	319 (94.1)	273 (80.5)	319 (94.1)
Unilateral	30 (14.9)	20 (10)	13 (6.5)	14 (7)	11 (5.5)	40 (13.2)	30 (9.9)	19 (6.3)	13 (4.3)	45 (13.3)	31 (9.1)	15 (4.4)	45 (13.3)	15 (4.4)
Bilateral	14 (9)	6 (3)	7 (3.5)	6 (3)	4 (2)	18 (6.0)	11 (3.6)	10 (3.3)	5 (1.7)	21 (6.2)	14 (4.1)	5 (1.5)	21 (6.2)	5 (1.5)

Features		T0-T6-T12-T18-T24					T0-T6-T12-T24				T0-T6-T24			T0-T24	
		T0	T6	T12	T18	T24	T0	T6	T12	T24	T0	T6	T24	T0	T24
Small foot j involvement (Y/N)															
	Yes	18 (9)	26 (13)	20 (10)	20 (10)	15 (7.5)	58 (19.2)	41 (13.6)	29 (9.6)	18 (6)	66 (19.5)	45 (13.3)	20 (5.9)	66 (19.5)	20 (5.9)
	No	183 (91)	175 (87)	181 (90)	181 (90)	186 (92.5)	244 (80.8)	261 (86.4)	273 (90.4)	284 (94)	273 (80.5)	294 (86.7)	319 (94.1)	273 (80.5)	319 (94.1)
NSAIDS															
	Yes	146 (72.6)	54 (26.9)	26 (12.9)	12 (6)	18 (9)	224 (74.2)	83 (27.5)	38 (12.6)	26 (8.6)	253 (74.6)	243 (71.7)	31 (9.1)	253 (74.6)	31 (9.1)
	No	55 (27.4)	147 (73.1)	175 (87.1)	189 (94)	183 (91)	78 (25.8)	219 (72.5)	264 (87.4)	276 (91.4)	86 (25.4)	96 (28.3)	308 (90.9)	86 (25.4)	308 (90.9)
Intraarticular GCs															
	Yes	18 (9)	129 (64.2)	43 (21.4)	31 (15.4)	16 (8)	22 (7.3)	181 (59.9)	64 (21.2)	27 (8.9)	24 (7.1)	202 (59.6)	32 (9.4)	24 (7.1)	32 (9.4)
	No	183 (91)	72 (35.8)	158 (78.6)	170 (84.6)	185 (92)	280 (92.7)	121 (40.1)	238 (78.8)	275 (91.1)	315 (92.9)	137 (40.4)	307 (90.6)	315 (92.9)	307 (90.6)
Systemic GCs															
	Yes	13 (6.5)	32 (15.9)	18 (9)	9 (4.5)	10 (5)	20 (6.6)	48 (15.9)	29 (9.6)	14 (4.6)	23 (6.8)	57 (16.8)	16 (4.7)	23 (6.8)	16 (4.7)
	No	188 (93.5)	169 (84.1)	183 (91)	192 (95.5)	191 (95)	282 (93.4)	254 (84.1)	273 (90.4)	288 (95.4)	316 (93.2)	282 (83.2)	323 (95.3)	316 (93.2)	323 (95.3)
Methotrexate															
	Yes	0 (0)	99 (49.3)	114 (56.7)	81 (40.3)	78 (38.8)	0 (0)	158 (52.3)	175 (57.9)	182 (60.3)	0 (0)	176 (51.9)	206 (60.8)	0 (0)	206 (60.8)
	No	201 (100)	102 (50.7)	87 (43.3)	120 (59.7)	123 (61.2)	302 (100)	144 (47.7)	127 (42.1)	120 (39.7)	339 (100)	163 (48.1)	133 (39.2)	339 (100)	133 (39.2)

	T0-T6-T12-T18-T24					T0-T6-T12-T24				T0-T6-T24			T0-T24	
Features	T0	T6	T12	T18	T24	T0	T6	T12	T24	T0	T6	T24	T0	T24
Sulfasalazine														
Yes	0 (0)	2 (1)	1 (0.5)	2 (1)	2 (1)	0 (0)	2 (0.7)	1 (0.3)	2 (0.7)	0 (0)	2 (0.6)	2 (0.6)	0 (0)	2 (0.6)
No	201 (100)	199 (99)	200 (99.5)	199 (99)	199 (99)	302 (100)	300 (99.3)	301 (99.7)	300 (99.3)	339 (100)	339 (100)	337 (99.4)	339 (100)	337 (99.4)
Cyclosporine														
Yes	0 (0)	0 (0)	2 (1)	1 (0.5)	2 (1)	0 (0)	0 (0)	2(0.7)	2(0.7)	0 (0)	0 (0)	2(0.6)	0 (0)	2(0.6)
No	201 (100)	201 (100)	199 (99)	200 (99.5)	199 (99)	302 (100)	302 (100)	300 (99.3)	300 (99.3)	339 (100)	339 (100)	337 (99.4)	339 (100)	337 (99.4)
Etanercept														
Yes	0 (0)	8 (4)	16 (8)	26 (12.9)	28 (13.9)	0 (0)	12 (4.0)	27 (8.9)	43 (14.2)	0 (0)	13 (3.8)	46 (13.6)	0 (0)	46 (13.6)
No	201 (100)	193 (96)	185 (92)	175 (87.1)	173 (86.1)	302 (100)	290 (96)	275 (91.1)	259 (85.8)	339 (100)	326 (96.2)	293 (86.4)	339 (100)	293 (86.4)
Tocilizumab														
Yes	0 (0)	0 (0)	2 (1)	4 (2)	6 (3)	0 (0)	0 (0)	5 (1.7)	12 (4)	0 (0)	0 (0)	14 (4.1)	0 (0)	14 (4.1)
No	201 (100)	201 (100)	199 (99)	197 (98)	195 (97)	302 (100)	302 (100)	297 (98.3)	290 (96)	361 (100)	361 (100)	325 (95.9)	361 (100)	325 (95.9)
Tocilizumab														
Yes	0 (0)	0 (0)	1 (0.5)	2 (1)	2 (1)	0 (0)	0 (0)	1 (0.3)	2 (0.7)	0 (0)	0 (0)	3 (0.8)	0 (0)	3 (0.8)
No	201 (100)	201 (100)	200 (99.5)	199 (99)	199 (99)	302 (100)	302 (100)	301 (99.7)	300 (99.3)	339 (100)	339 (100)	337 (99.4)	339 (100)	337 (99.4)
Anakinra														
Yes	0 (0)	3 (1.5)	2 (1)	1 (0.5)	2 (1)	0 (0)	3 (1.0)	2 (0.7)	3 (1)	0 (0)	5 (1.5)	5 (1.5)	0 (0)	5 (1.5)
No	201 (100)	198 (98.5)	199 (99)	200 (99.5)	199 (99)	302 (100)	299 (99)	300 (99.3)	299 (99)	339 (100)	334 (98.5)	334 (98.5)	339 (100)	334 (98.5)

	T0-T6-T12-T18-T24					T0-T6-T12-T24				T0-T6-T24			T0-T24	
**Features**	**T0**	**T6**	**T12**	**T18**	**T24**	**T0**	**T6**	**T12**	**T24**	**T0**	**T6**	**T24**	**T0**	**T24**
Canakinumab														
Yes	0 (0)	2 (1)	2 (1)	2 (1)	1 (0.5)	0 (0)	2 (0.7)	2 (0.7)	1 (0.3)	0 (0)	2 (0.6)	2 (0.6)	0 (0)	2 (0.6)
No	201 (100)	199 (99)	199 (99)	199 (99)	200 (99.5)	302 (100)	300 (99.3)	300 (99.3)	301 (99.7)	339 (100)	337 (99.4)	337 (99.4)	339 (100)	337 (99.4)
Baricitinib														
Yes	0 (0)	0 (0)	0 (0)	0 (0)	0 (0)	0 (0)	0 (0)	0 (0)	1 (0.3)	0 (0)	0 (0)	1 (0.3)	0 (0)	1 (0.3)
No	201 (100)	201 (100)	201 (100)	201 (100)	201 (100)	302 (100)	302 (100)	302 (100)	301 (99.7)	339 (100)	339 (100)	338 (99.7)	339 (100)	338 (99.7)
Infliximab														
Yes	0 (0)	0 (0)	0 (0)	0 (0)	0 (0)	0 (0)	0 (0)	0 (0)	1 (0.3)	0 (0)	0 (0)	1 (0.3)	0 (0)	1 (0.3)
No	201 (100)	201 (100)	201 (100)	201 (100)	201 (100)	302 (100)	302 (100)	302 (100)	301 (99.7)	339 (100)	339 (100)	338 (99.7)	339 (100)	338 (99.7)
Number of medications														
0	171 (85.1)	16 (8)	62 (30.8)	56 (27.9)	57 (28.4)	261 (86.4)	28 (9.3)	89 (29.5)	87 (28.8)	293 (86.4)	35 (10.3)	95 (28)	293 (86.4)	95 (28)
1	29 (14.4)	105 (52.2)	90 (44.8)	100 (49.8)	103 (51.2)	40 (13.2)	160 (53)	138 (45.7)	154 (51)	45 (13.3)	173 (51)	174 (51.3)	45 (13.3)	174 (51.3)
2	1 (0.5)	68 (33.8)	37 (18.4)	38 (18.9)	35 (17.4)	1 (0.3)	97 (32.1)	57 (18.9)	51 (16.9)	1 (0.3)	110 (32.4)	59 (17.4)	1 (0.3)	59 (17.4)
3	0 (0)	11 (5.5)	11 (5.5)	6 (3)	5 (2.5)	0 (0)	16 (5.3)	16 (5.3)	7 (2.3)	0 (0)	20 (5.9)	8 (2.4)	0 (0)	8 (2.4)
4	0 (0)	1 (0.5)	1 (0.5)	1 (0.5)	1 (0.5)	0 (0)	1 (0.3)	2 (0.7)	1 (0.3)	0 (0)	1 (0.3)	1 (0.3)	0 (0)	1 (0.3)
5	0 (0)	0 (0)	0 (0)	0 (0)	0 (0)	0 (0)	0 (0)	0 (0)	2 (0.7)	0 (0)	0 (0)	2 (0.6)	0 (0)	2 (0.6)

Features		T0-T6-T12-T18-T24					T0-T6-T12-T24				T0-T6-T24			T0-T24	
		T0	T6	T12	T18	T24	T0	T6	T12	T24	T0	T6	T24	T0	T24
ID															
	0	201 (100)	37 (18.4)	138 (68.7)	127 (63.2)	119 (59.2)	302 (100)	233 (77.2)	196 (64.9)	174 (57.6)	339 (100)	256 (75.5)	199 (55.1)	339 (100)	199 (55.1)
	1	0 (0)	164 (81.6)	66 (31.3)	74 (36.8)	82 (40.8)	0 (0)	69 (22.8)	106 (35.1)	128 (42.4)	0 (0)	83 (24.5)	162 (44.9)	0 (0)	162 (44.9)

ANA = Antinuclear antibody positive ; RF= Rheumatoid Factor; HLAB27= human leukocyte antigen B27 ; PhGA= physician's global assessment ; AJC= Active joint count ; ESR = erythrocyte sedimentation rate ; CRP= C-reactive protein ; R = right; L = left; ID= inactive disease ; NSAIDs = nonsteroidal anti-inflammatory drugs; TMJ = temporomandibular joint; J= joints.
