# Supplementary material for: Forecasting Achievement of Inactive Disease in Juvenile Idiopathic Arthritis with Artificial Intelligence
Source: Children (Basel). 2025 Jun 7;12(6):741. doi: 10.3390/children12060741 (PMC12191878; doi:10.3390/children12060741)
Supplement: Supplementary file 1 [file children-12-00741-s001.zip › Supplementary Table S3.pdf]

Supplementary Table 3. Forecasting MCC of additional AI algorithms coupled with the MLforecast model based on all clinical features and selected time points

Dataset	Penalized regression		KNN		SVM	
	Training set	Testing set	Training set	Testing set	Training set	Testing set
T_0- T_6 -T_{12}-T_{18}-T_{24}	0.61	0.43	0.67	0.20	0.70	0.53
T_0- T_6 -T_{12}-T_{24}	0.54	0.66	0.49	0.40	0.62	0.59
T_0- T_6 -T_{24}	0.51	0.42	0.45	0.31	0.56	0.50
T_0-T_{24}	0.0	0.0	0.0	0.0	0.0	0.0

MCC =Matthews Correlation Coefficient. 0≤ MCC≤0.19 (Very low), 0.2≤ MCC≤0.39 (low), 0.4≤ MCC≤0.59 (Moderate), 0.6≤ MCC≤0.79 (high), and 0.8≤ MCC≤1.0 (very high). Performance of the penalized logistic regression models were obtained with the following hyperparameters for each dataset: T0-T6-T12-T18-T24: 'C': 43.5674376783705, 'penalty': 'l1'; T0-T6-T12-T24: 'C': 0.16064293007278221, 'penalty': 'l1'; T0-T6-T24: 'C': 43.699018950495436, 'penalty': 'l2'. Performance of the KNN models were obtained with the following hyperparameters for each dataset: T0-T6-T12-T18-T24: 'n_neighbors': 12, 'weights': 'distance', 'p': 1; T0-T6-T12-T24: 'n_neighbors': 13, 'weights': 'distance', 'p': 1; T0-T6-T24: 'n_neighbors': 21, 'weights': 'distance', 'p': 1. Performance of the SVM models were obtained with the following hyperparameters for each dataset: T0-T6-T12-T18-T24: 'C': 56.57725665849967, 'kernel': 'poly', 'gamma': 0.07503472680994534,'degree': 2; T0-T6-T12-T24: 'C': 0.17124901389019045, 'kernel': 'poly', 'gamma': 0.0572365475951005, 'degree': 5; T0-T6-T24: 'C': 1.517622900189301, 'kernel': 'poly','gamma': 0.6440624282492913, 'degree': 2. For T0-T24, the data contains only one class and tuning rejected the selection of any hyperparameters.

KNN: K-Nearest Neighbor. SVM: Support Vector Machine
